# Supplementary figures and images for: Modulations of local synchrony over time lead to resting-state functional connectivity in a parsimonious large-scale brain model
Source: PLoS One. 2022 Oct 26;17(10):e0275819. doi: 10.1371/journal.pone.0275819 (PMC9604991; doi:10.1371/journal.pone.0275819)

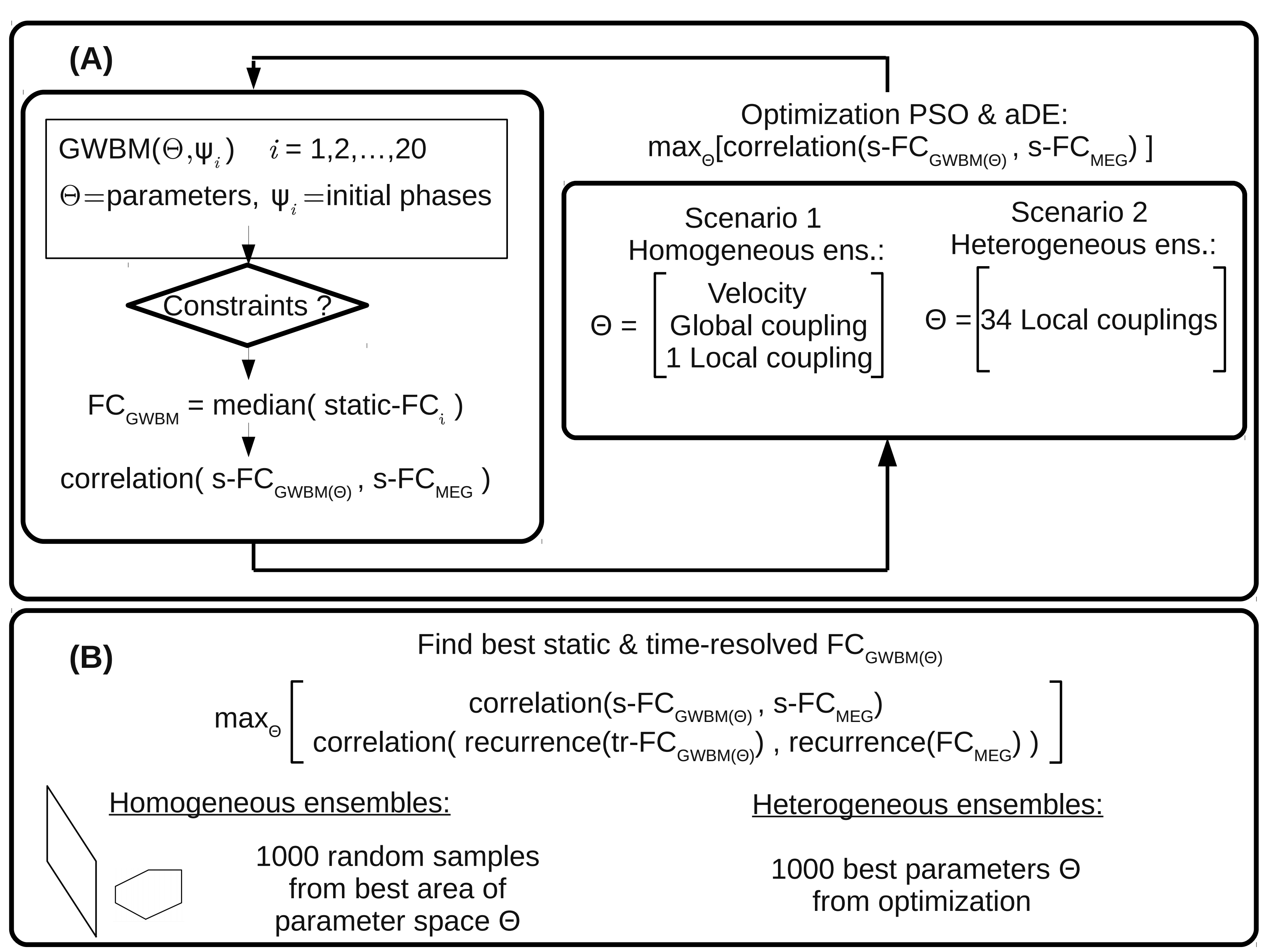

Supplement: S1 Fig — (A) Optimization of parameters for the first (homogeneous ensembles) or the second scenario (heterogeneous ensembles). (B) Simulations to find the parameters that are able to predict static FC and time-resolved FC. (TIF) [file pone.0275819.s002.tif]

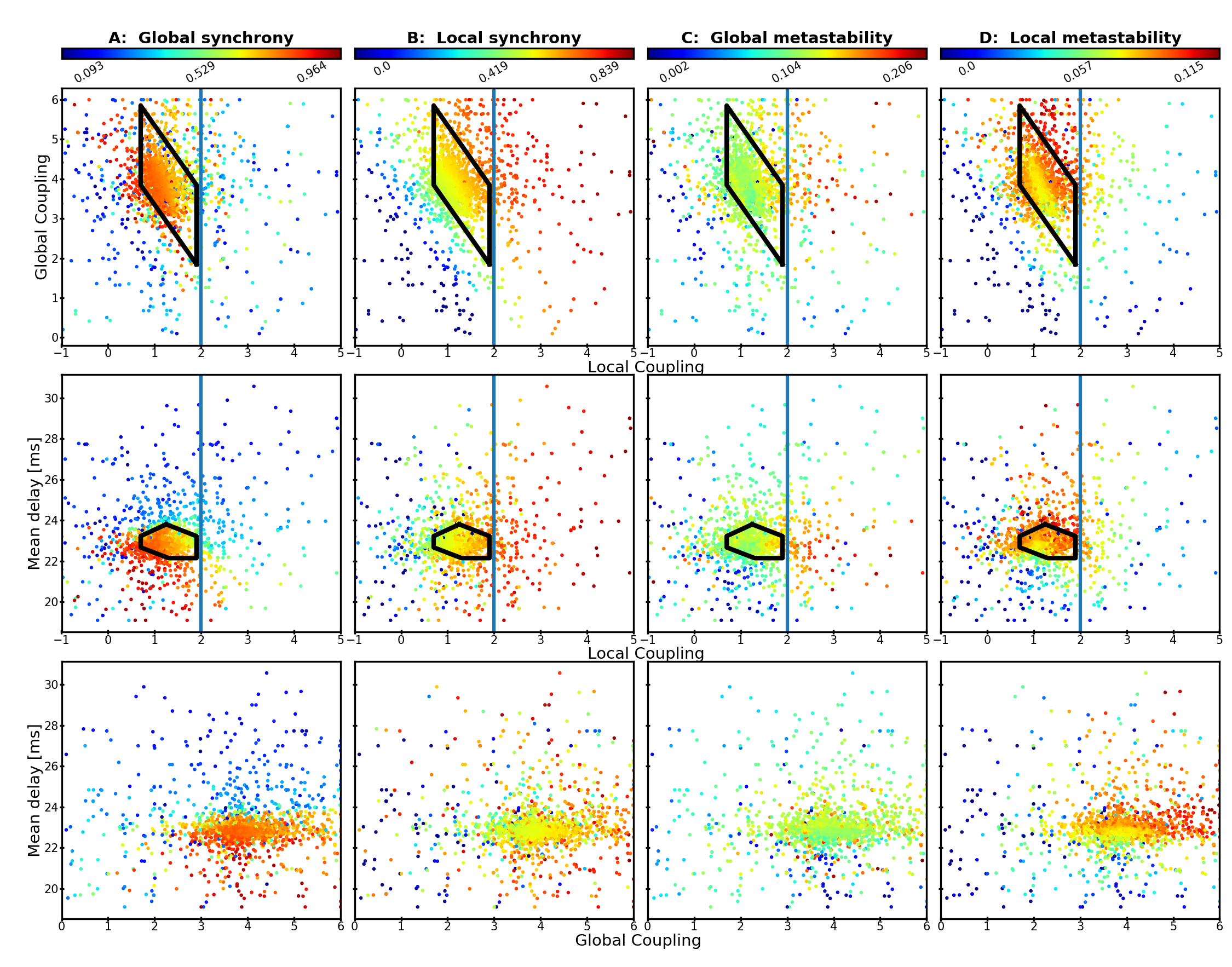

Supplement: S2 Fig — Each dot corresponds to one combination of parameters (x-y axis) during the optimization. Each column corresponds to a different dynamical feature. (A) Global synchrony averaged over time. (B) Local synchrony averaged over ensembles first and then over time. (C) Global metastability (D) Local metastability averaged over ensembles. For each metric, each value corresponds to the median obtained across 20 simulations computed with different initial conditions. Vertical blue lines indicate the critical local couplings of the ensembles, Lc. The black areas indicate the region of the parameter space within which time-resolved functional connectivity was analysed. (TIF) [file pone.0275819.s003.tif]

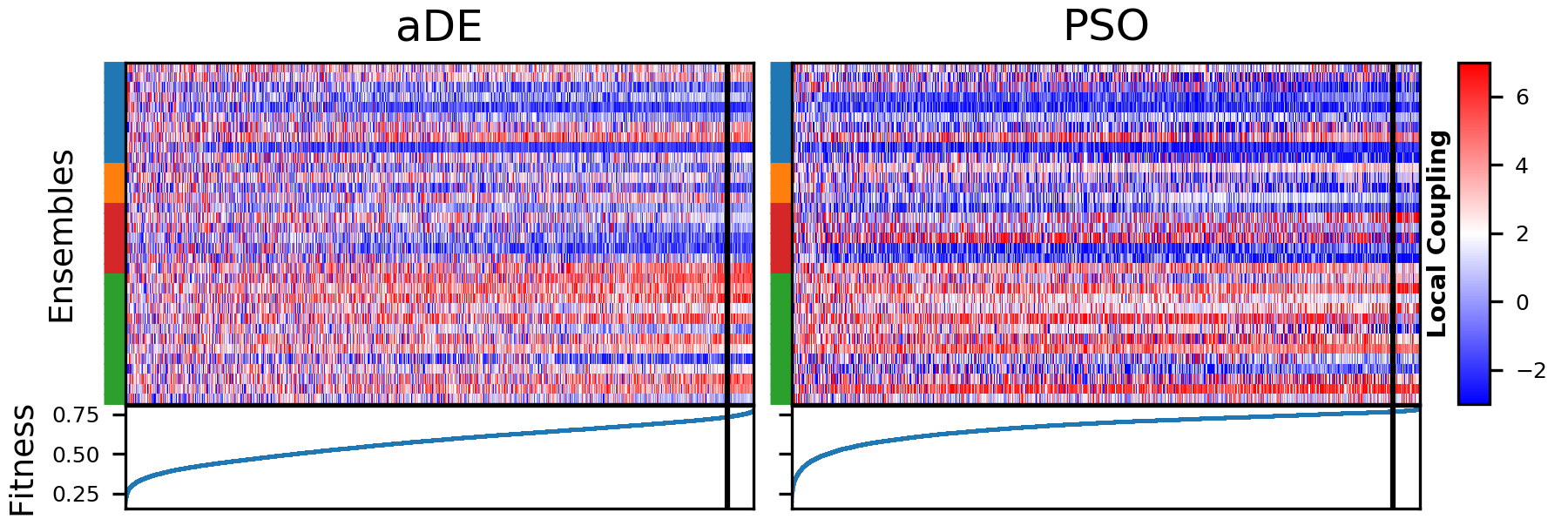

Supplement: S3 Fig — Each row inside the upper panel depicts the local couplings of one ensemble. The columns correspond to iterations of the respective optimizer (aDE and PSO). The brain lobe associated with each ensemble is color coded on the left (same as Fig 2). The fitness (Pearson correlation between MEG and simulated sFC) of each iteration is at the bottom of the panels. trFC was evaluated at the parameters to the right of the black vertical line. (TIF) [file pone.0275819.s004.tif]

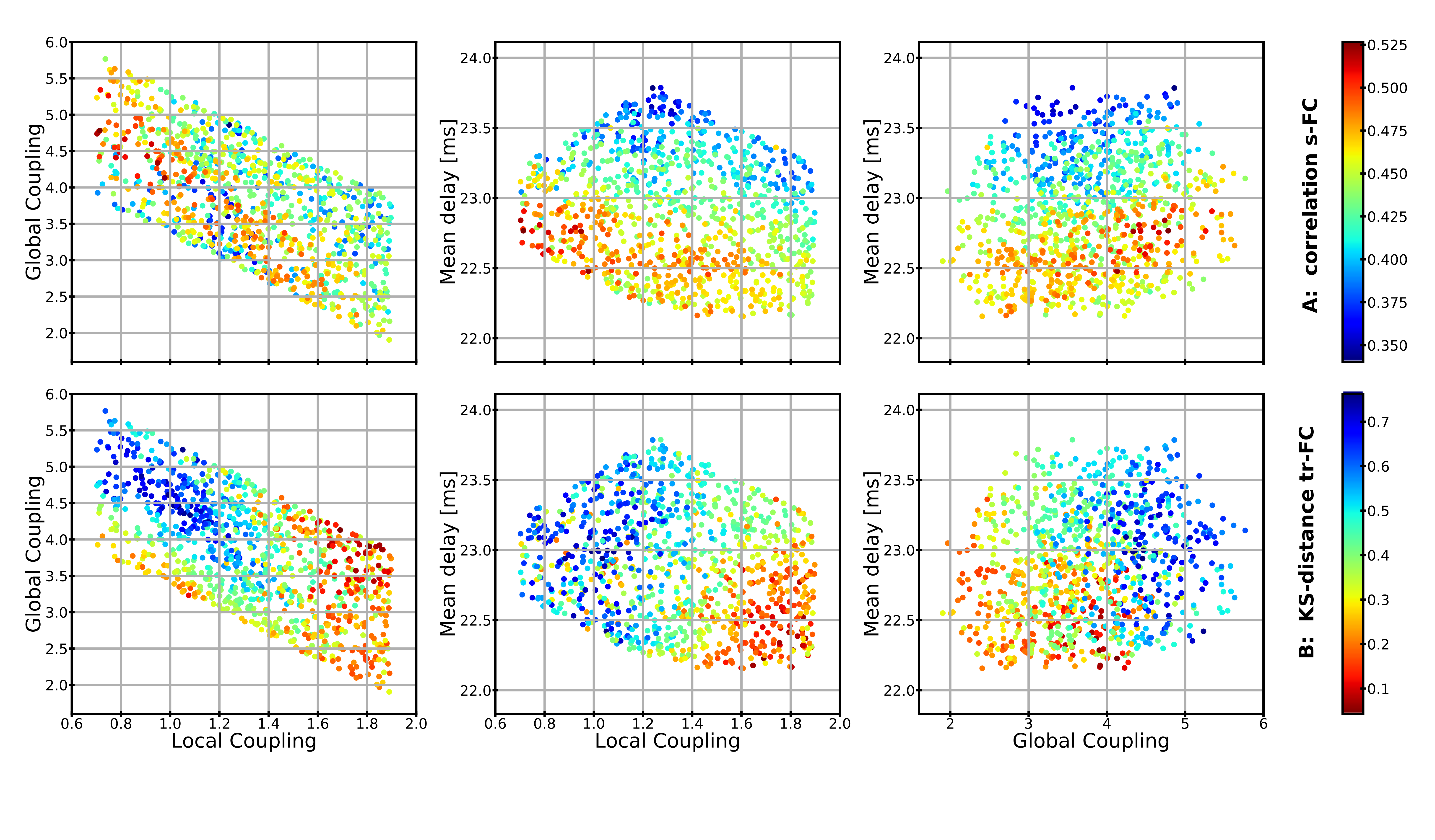

Supplement: S4 Fig — Each dot corresponds to one combination of parameters–global coupling, local coupling, and mean delay. The parameters are restricted to the area that gave the best sFC predictions during the optimization (black area in Fig 2). (sFC, first row) correlation between the simulated and MEG sFC. (trFC, second row) KS-distance between the histograms (simulated vs. MEG data) built from the recurrence of sFC recurrence over a 15-second sliding window with 12-second overlap. Each dot is the median of 30 simulations with identical parameters and different initial conditions. (TIF) [file pone.0275819.s005.tif]

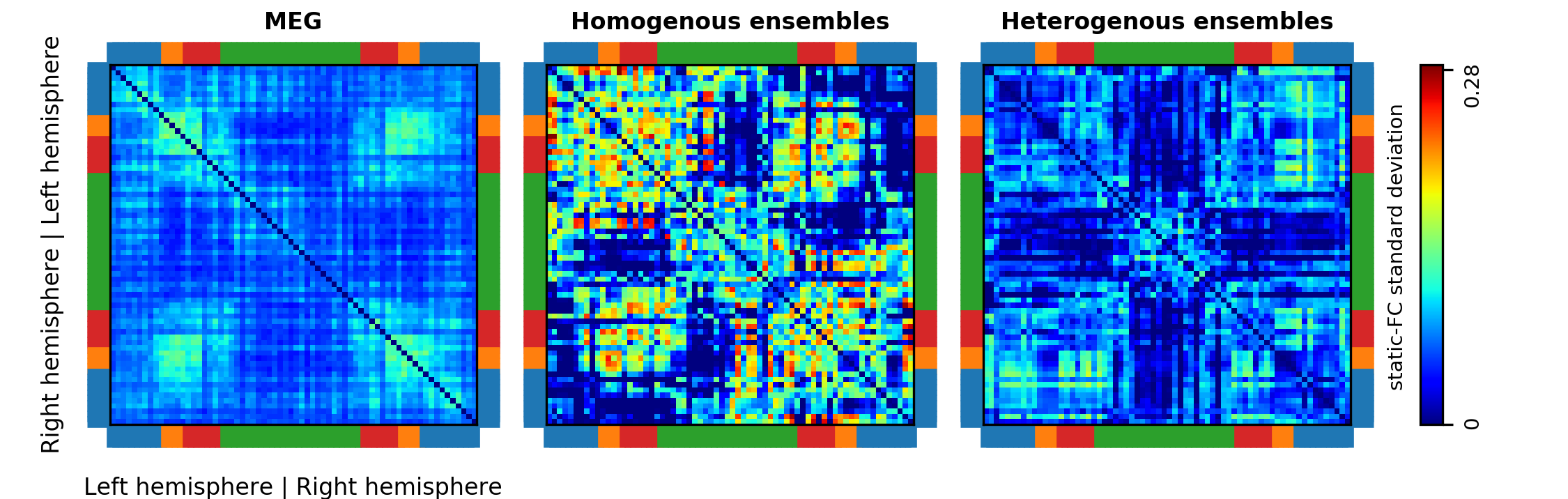

Supplement: S5 Fig — (Left) Standard deviation of the FC over subjects (Middle, model with homogeneous ensembles) Standard deviation of the FC from simulations with identical parameters but different initial conditions. (Right, model with heterogeneous ensembles) Standard deviation of the FC from simulations with identical parameters but different initial conditions. (TIF) [file pone.0275819.s006.tif]

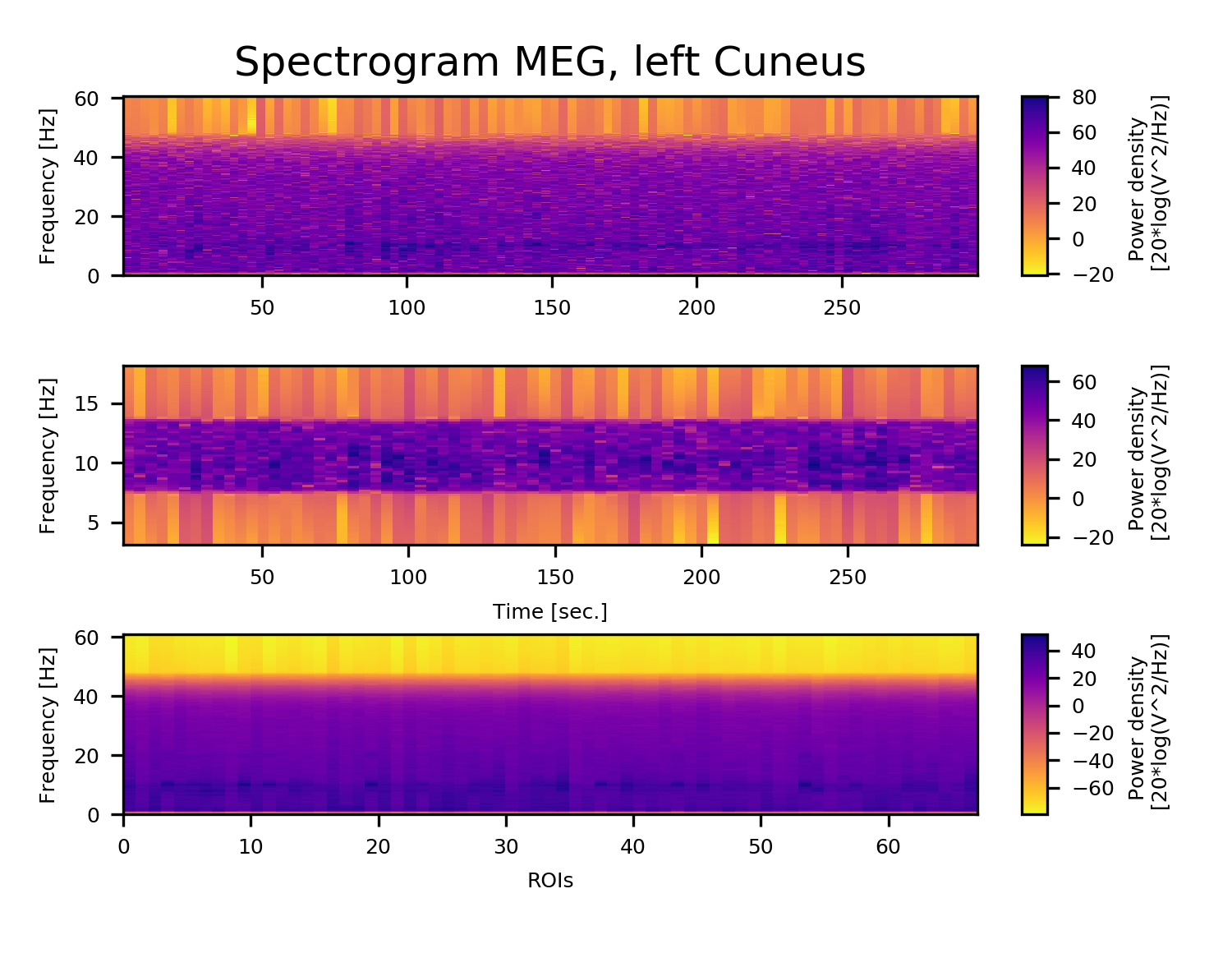

Supplement: S6 Fig — (Above) Broadband activity with a low-pass filter at 48 Hz. (Middle) Band-pass filtered alpha-band activity. (Bottom) Welch periodogram of broadband activity in all ROIs. (TIF) [file pone.0275819.s007.tif]

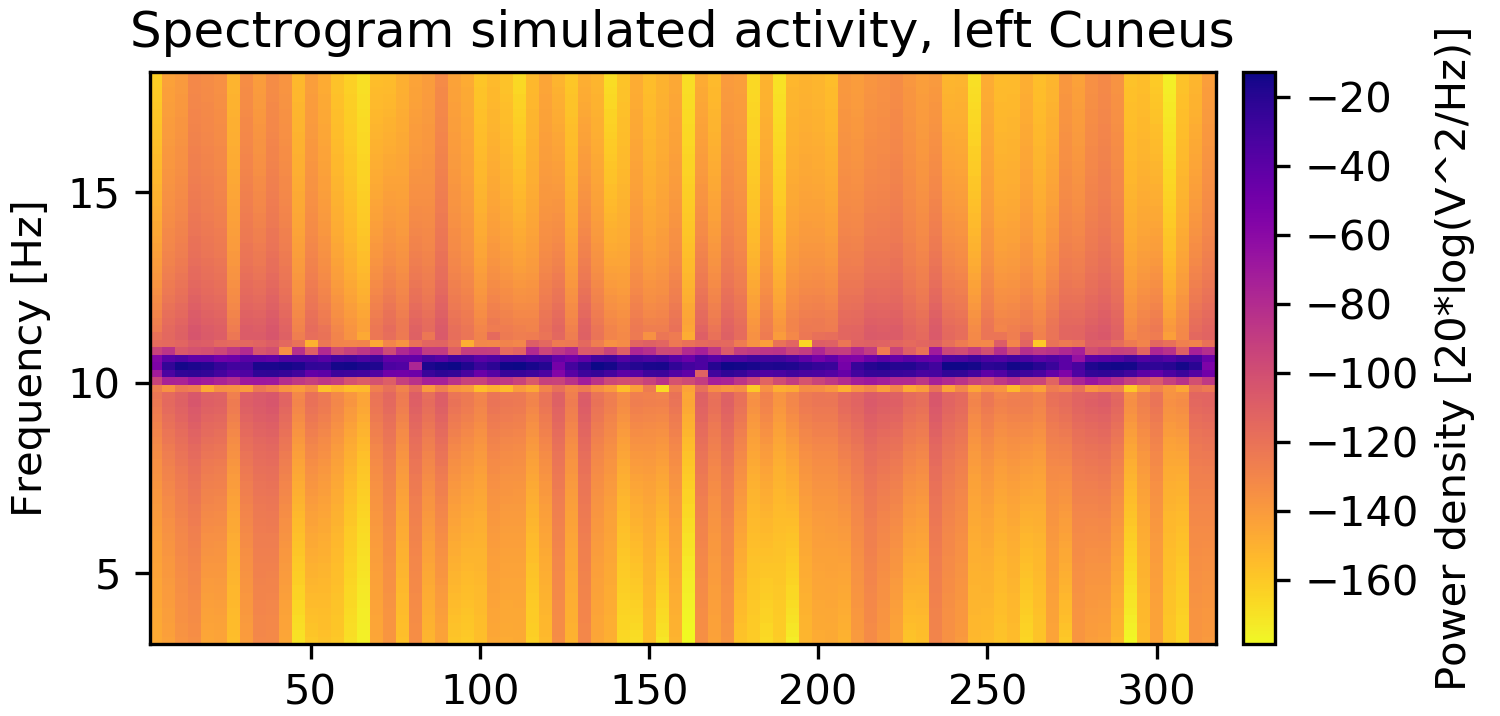

Supplement: S7 Fig — The parameters are the same parameters as Figs 2 and 5 for heterogeneous ensembles. (TIF) [file pone.0275819.s008.tif]
